# Terpenoid Compositions and Botanical Origins of Late Cretaceous and Miocene Amber from China

**DOI:** 10.1371/journal.pone.0111303

**Published:** 2014-10-29

**Authors:** Gongle Shi, Suryendu Dutta, Swagata Paul, Bo Wang, Frédéric M. B. Jacques

**Affiliations:** 1 State Key Laboratory of Palaeobiology and Stratigraphy, Nanjing Institute of Geology and Palaeontology, Chinese Academy of Sciences, Nanjing, China; 2 Department of Earth Sciences, Indian Institute of Technology Bombay, Mumbai, India; 3 Steinmann Institute, University of Bonn, Bonn, Germany; 4 Key Laboratory of Tropical Forest Ecology, Xishuangbanna Tropical Botanical Garden, Chinese Academy of Sciences, Mengla, China; Institute of Botany, China

## Abstract

The terpenoid compositions of the Late Cretaceous Xixia amber from Central China and the middle Miocene Zhangpu amber from Southeast China were analyzed by gas chromatography-mass spectrometry (GC-MS) to elucidate their botanical origins. The Xixia amber is characterized by sesquiterpenoids, abietane and phyllocladane type diterpenoids, but lacks phenolic abietanes and labdane derivatives. The molecular compositions indicate that the Xixia amber is most likely contributed by the conifer family Araucariaceae, which is today distributed primarily in the Southern Hemisphere, but widely occurred in the Northern Hemisphere during the Mesozoic according to paleobotanical evidence. The middle Miocene Zhangpu amber is characterized by amyrin and amyrone-based triterpenoids and cadalene-based sesquiterpenoids. It is considered derived from the tropical angiosperm family Dipterocarpaceae based on these compounds and the co-occurring fossil winged fruits of the family in Zhangpu. This provides new evidence for the occurrence of a dipterocarp forest in the middle Miocene of Southeast China. It is the first detailed biomarker study for amber from East Asia.

## Introduction

Amber is fossilized natural resin that was produced by secretary cells of ancient plants. It is known as a source for jewelry and also as an effective preservation medium for fossil insects and soft-bodied microorganisms [1]–[4]. Resins are polymerized from a broad range of isoprenoid compounds including primarily terpenoids, carboxylic acids and associated alcohols [5], [6]. The function of resin is not clearly understood, but is considered to protect plants from invasion by fungi and insects after injury [7]–[9]. Terpenoids are amongst the most diverse plant natural products, with about 25,000 known compounds and are often diagnostic for certain plant groups [10]. Albeit various chemical transformations during burial, the terpenoids in fossil resins and fossil plant remains often retain their characteristic basic structural skeletons and can thus be used as biomarkers for botanical origins of the fossils [5], [11]. Based on compositions of terpenoids, for example, the Early Cretaceous Burmese amber was considered derived from the conifer family Pinaceae [12]; the early Eocene Cambay amber of India from the tropical angiosperm family Dipterocarpaceae [13]–[15], and the Eocene Baltic amber of Europe from the conifer family Sciadopityaceae [6]. Other plant families from which amber was probably produced include Araucariaceae, Cupressaceae *sensu lato* (including Cupressaceae *sensu stricto* and Taxodiaceae), Leguminosae, Burseraceae, Hamamelidaceae, Combretaceae and the extinct conifer family Cheirolepidiaceae [2], [16].

**Figure 1:**
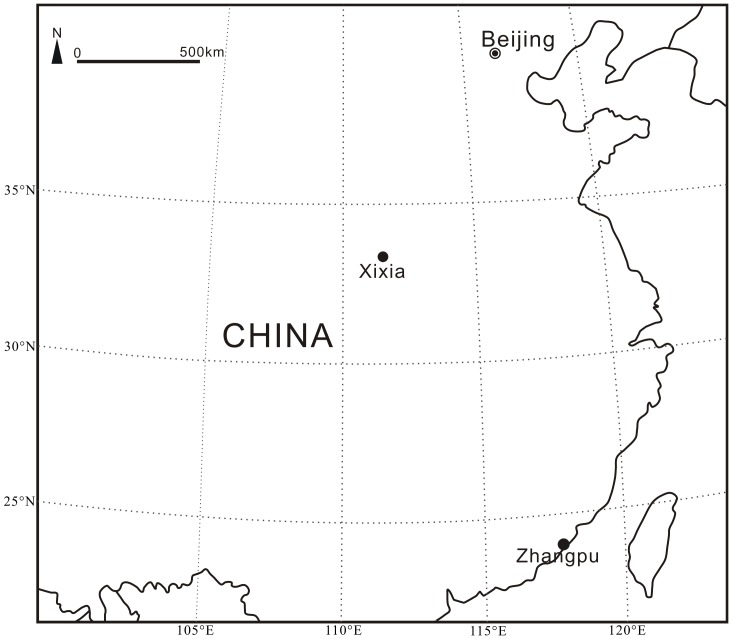
Map showing the locations of Xixia and Zhangpu where the studied amber was collected.

**Figure 2:**
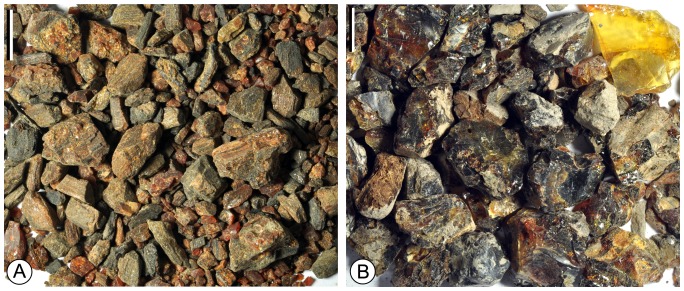
Photos of Late Cretaceous Xixia amber (A) and middle Miocene Zhangpu amber (B).

As a kind of traditional Chinese medicine that could calm nerve, amber has been known and collected for a long history in China. Although amber has been reported from several sites in China, most of these sites were known based on very little amber pieces [17], [18]. Only in three sites amber has been extensively collected by local people, including Fushun in Northeast China, Xixia in Central China, and Zhangpu in Southeast China (Fig. 1). The early Eocene Fushun amber is well-known for containing a diverse assemblage of fossil insects and plant remains, and has been studied in entomological, gemological and geochemical aspects [4], [19]. The terpenoid compositions indicate that the Fushun amber was derived from the conifer family Cupressaceae [4]. In contrast to the Fushun amber, little attention has been paid to the Late Cretaceous Xixia amber (Fig. 2A) and middle Miocene Zhangpu amber (Fig. 2B) probably because they are minute or fragile and thus not suitable for making jewelry. In this paper we investigated the terpenoid compositions of the Xixia and Zhangpu amber and explored its botanical affinities based on biomarkers.

## Materials and Methods

### Ethics Statement

The collection of amber under study was permitted by the local governments of Xixia County, Henan Province and Zhangpu County, Fujian Province. The field trip was done in non-National Nature Reserves and non-private areas. We did not violate the Chinese fossil collection and mining laws and management regulations.

### Localities and geological setting

**Figure 3:**
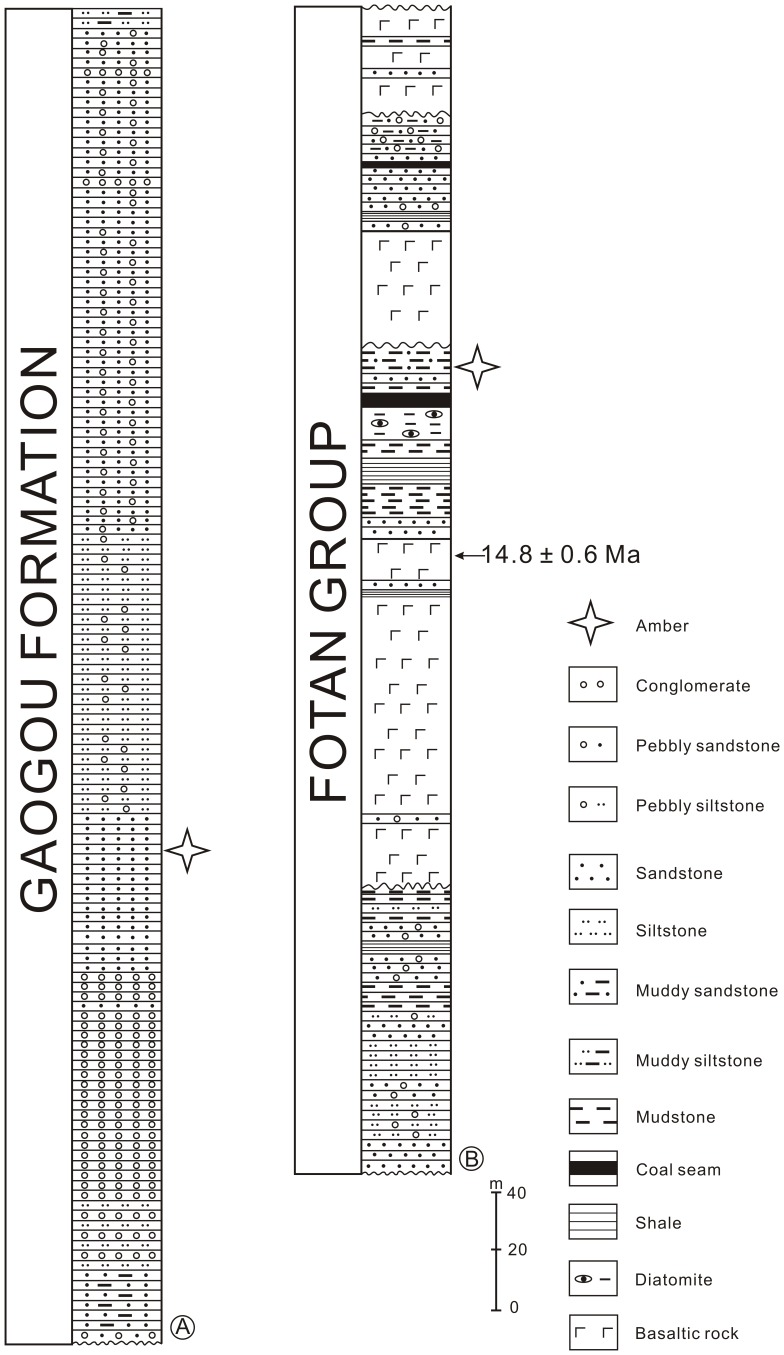
Generalized stratigraphic sections of the Gaogou Formation in Xixia (A) and Fotan Group in Zhangpu (B). The stratigraphic position of the amber (indicated by four pointed star) and radiometric age of the basaltic rock samples are shown.

The Late Cretaceous Xixia amber was collected from the Gaogou Formation at Wuliqiao Town (33°18′39′′N, 111°28′48′′E), Xixia County, Henan Province, Central China (Fig. 1). The Gaogou Formation is amongst the non-marine Late Cretaceous red beds in Xixia Basin that are well-known for abundant well-preserved dinosaur eggs [20], [21]. The formation is composed of brownish red calcareous siltstones, fine to coarse-grained sandstones, pale sandy conglomerates, purplish- and brownish-red siltstones and pebble-bearing sandstones intercalated with silty mudstones (Fig. 3A) [20]. It is assigned to Late Cretaceous, most probably Coniacian-Campanian, constrained by studies of fossil bivalve [22] and ostracoda assemblages [23]. Plant macrofossils and pollen have not been reported in the Gaogou Formation. Xixia possesses the currently known largest amber deposits in China [18]. The Xixia amber is preserved as lenses in the fine-grained sandstones in the middle part of the Gaogou Formation (Fig. 3A). The amber is yellow, brown, brownish yellow, brownish red in color, the single amber pieces are usually minute in dimension (Fig. 2A). So far no insects have been reported from the Xixia amber.

The Miocene Zhangpu amber was collected from the Fotan Group at Qianting Town (24°16′03′′N, 117°59′01′′E), Zhangpu County, Fujian Province, Southeast China (Fig. 1). The Fotan Group is mainly distributed in the coastal areas of southeastern Fujian, as well as in Mingxi and Ninghua County of western Fujian [24]. It consists of arenaceous conglomerate rocks, sandstones, sandy mudstones, mudstones, lignite and diatomite, with interbedded three layers of basaltic rocks derived from several episodes of volcanic activity during the Neogene (Fig. 3B) [24]. The ^40^Ar −^39^Ar dating of the basaltic rocks in the middle part of the Fotan Group rendered an age of 14.7–14.9±0.6 Ma (Fig. 3B) [25], belonging to the Langhian Stage (middle Miocene), consistent with the study of palynological assemblage of the group [24]. The Fotan Group in Zhangpu County yields abundant well-preserved plant leaf and fruit fossils, including Clusiaceae, Dipterocarpaceae, Fagaceae, Hamamelidaceae, Lauraceae, Leguminosae and Moraceae. The plant fossil assemblage of the Fotan Group indicates a middle Miocene dipterocarp forest and probable tropical rain forest in Southeast China [26], [27]. The Zhangpu amber is preserved in blue-grey sandy mudstone, or sometimes *in situ* in lignified fossil wood. It is yellow, brown to brownish red in color, the single pieces of Zhangpu amber can be big but they are extremely fragile (Fig. 2B). Although neither insects nor plant microfossils have been reported from the Zhangpu amber, it is considered potential source for paleontological studies.

### Repository

Approximately 200 g Xixia amber and 3 kg Zhangpu amber were collected and deposited permanently in Nanjing Institute of Geology and Palaeontology, Chinese Academy of Sciences. The Xixia amber pieces were preserved in one vial and assigned one registered number, PB21517; the Zhangpu amber pieces were assigned PB21518.

### Gas Chromatography-Mass Spectrometry (GC-MS)

Amber fragments were extracted with a dichloromethane and methanol (ratio 9∶1) mixture for one hour. The total extracts were analyzed by gas chromatography-mass spectrometry (GC-MS). The GC-MS analysis was performed on an Agilent 5975 mass spectrometer interfaced to a 7890 gas chromatograph. Extracts were analyzed on HP-5 MS fused silica (30 m × 0.25 mm i.d., × 0.25 µm film thickness) GC column. Helium was used as carrier gas at a flow rate of 1 ml/min. The initial GC oven temperature was held at 40°C for 5 minutes and then ramped to 310°C at a rate of 4°C/min. The mass spectrometer detector was programmed as EI mode with ionization energy 70 eV. The samples were analyzed in a full scan mode (mass range 50–600 dalton). The data processing was taken by Chemstation software and the identification of compounds was carried out based on their elution pattern and the comparison of mass spectra with published literatures.

## Results and Discussions

### Late Cretaceous Xixia amber (Fig. 4; Table 1)

**Figure 4:**
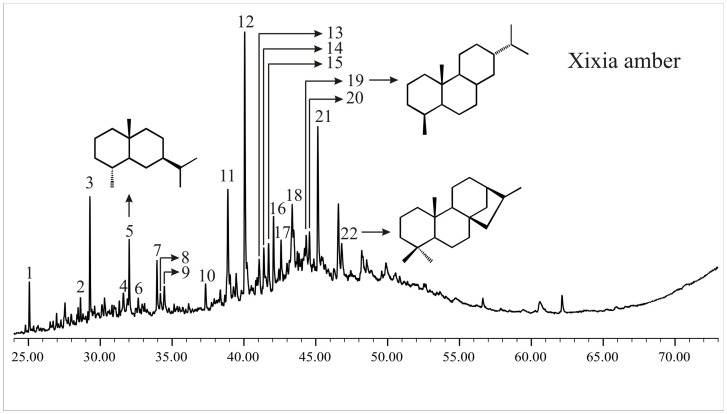
Total ion chromatogram of the Late Cretaceous Xixia amber from GC-MS analysis. The identified peaks are listed in Table 1.

The total ion chromatogram from the GC-MS analysis of the Xixia amber is characterized by the distribution of sesquiterpenoids and diterpenoids (Fig. 4; Table 1). The major sesquiterpenoids are drimane, homodrimane, 1,1,6-trimethyl-1,2,3,4-tetrahydronaphthalene, 4β-eudesmane, ionene and some unknown C_13_-C_17_ sesquiterpenoids with base peak 109 (Fig. 4; Table 1). Most of the diterpenoids belong to abietane class and these are 16,17,19-trisnorabietane, bisnorabietane, trisnorabieta-8,11,13-triene, norabietane and fichtelite. Besides, tricyclic rimuane and tetracyclic phyllocladane are also abundantly present in the solvent extract of the Xixia amber. Phenolic abietanes such as totarol, ferruginol, sugiol were not detected. The molecular compositions suggest that the Xixia amber is most likely derived from the conifer family Araucariaceae.

**Table 1:** Major compounds identified from the Late Cretaceous Xixia amber.

Peak number	Compound Name	Base peak	Molecular ion
1	Unknown C_13_sesquiterepnoid	109	180
2	Unknown C_14_sesquiterpenoid	109	194
3	Unknown C_14_sesquiterpenoid	109	194
4	Drimene+1,1,6-Trimethyl-1,2,3,4-tetrahydronaphthalene	123,159	208,174
5	4β-Eudesmane	109	208
6	Ionene	173	188
7	C_15_sesquiterpenoid	109	206
8	Homodrimane	123	222
9	Unknown C_16_sesquiterpenoid+ Trimethyl naphthalene	109,155	220,170
10	C_17_ sesquiterpenoid	109	236
11	16,17,19-trisnorabietane	109	234
12	Bisnorabietane	109	248
13	Isomer of bisnorabietane	109	248
14	Trisnorabieta-8,11,13-triene	131	228
15	Norabietane	109	262
16	Possibly isomer of norabietane	109	262
17	Unknown C_18_diterpenoid	109	248
18	C_19_ Diterpenoid	109	260
19	Fichtelite	109	262
20	Possibly isomer of norabietane	95	262
21	Unknown C_20_diterpenoidpossibly rimuane	109	276
22	α- phyllocladane	123	274

Abietane type diterpenoids that are detected in the Xixia amber are characteristic biomarkers for gymnosperms, especially conifers [5]. These diterpenoids are derived from abietic acid which has been reported from almost all conifer families [5]. However, the presence of tetracyclic diterpenoids (e.g. phyllocladane) can exclude the contribution of Pinaceae to the Xixia amber since resins of extant Pinaceae differ from those of other extant conifer families in lacking phenolic abietanes and tetracyclic diterpenoids [5]. Moreover, the exclusive presence of labdane and abietane diterpenoids is also characteristic for extant Pinaceae [28] whereas labdane dericatives are not detected in the Xixia amber.

Derivatives of cedrane and cuparane, only reported from resins of extant Cupressaceae, are characteristic biomarkers for this conifer family [29]. These compounds are, however, not detected in the solvent extracts of Xixia amber, making a cupressaceous origin of the Xixia amber unlikely. Besides, the phenolic abietanes such as totarol, ferruginol, sugiol that are produced only by extant Cupressaceae and Podocarpaceae [5], [28], [30], are totally absent in the Xixia amber, further supporting the exclusion of a cupressaceous source for Xixia amber.

Tricyclic rimuane that is detected in the Xixia amber is derived from the Southern Hemisphere conifers Araucariaceae and Podocarpaceae [31] whereas phyllocladane is known from Araucariaceae, Podocarpaceae and Cupressaceae *sensu lato* [5]. The absence of phenolic abietanes limits the possibility of the podocarpaceous source of the Xixia amber and therefore it is most likely that the Xixia amber was derived from Araucariaceae. However, the contribution from Podocarpaceae cannot be completely ruled out.

Plant macrofossils (leaves, fruits, wood) and microfossils (pollen) that occur in or associated with amber are usually considered potential candidates for the botanical origins of the amber [1], [6], [14], [28], [32], [33]. Unfortunately, neither plant macrofossils nor pollen have been reported from the Gaogou Formation of Xixia Basin, so that it is impossible to explore the botanical source of Xixia amber based on co-occurring fossil plants currently. Further paleobotanical exploration in the Gaogou Formation may help clarify the origin of Xixia amber. In the following paragraphs we review briefly the biogeographical history of Araucariaceae and Podocarpaceae.

Araucariaceae contain three genera that are primarily in the Southern Hemisphere, with a disjunct distribution in Malaysia, Indonesia, Philippines, New Guinea, Australia, New Zealand, New Caledonia, Vanuatu, Fiji, Norfolk Island and southern South America [34]. Paleobotanical evidence indicates, however, the family had a wide distribution in both the Northern and Southern Hemispheres during the Mesozoic and disappeared in most parts of the Northern Hemisphere by the latest Cretaceous [35], [36]. Modern trees of Araucariaceae, especially the genus *Agathis* Salisb. are highly resinous [34] and araucariacean trees are suspected as a common source for amber all through the Mesozoic [32], [33], [37], [38]. It is of interest to note that amber had not become abundant until Early Cretaceous [16], when the Araucariaceae attained their greatest diversity and widest distribution in both the Northern and Southern Hemispheres [35].

Podocarpaceae consist of 18 extant genera that are distributed predominantly in the tropical and subtropical mountains of the Southern Hemisphere, and in the Northern Hemisphere extending northward to subtropical China, Japan, Mexico and the Caribbean Islands [34]. Different from Araucariaceae, living trees of Podocarpaceae are only slightly resinous [34], making the family unlikely a potential source for fossil resins. Biogeographically, Podocarpaceae are considered distributed essentially in Gondwana, or Gondwana-derived plates during the Cretaceous [39]. This appears not to support the occurrence of Podocarpaceae in the Cretaceous of East Asia and a podocarpaceous origin of the Late Cretaceous Xixia amber.

Cheirolepidiaceae, an extinct Mesozoic conifer family ranging from Late Triassic to Late Cretaceous [40], has been considered the botanical source of the Late Triassic amber of Italy based on the presence of *in situ* fossil resins within plant macrofossils of Cheirolepidiaceae, and the abundant occurrence of pollen and cuticles of this extinct family in the paleosol where the dispersed amber was buried [2]. Since that Cheirolepidiaceae was the most dominant conifer family through the Jurassic and Early Cretaceous, it has been considered potential candidate for the botanical origin of some Jurassic and Cretaceous amber [28], [41], [42]. Molecular composition analyses revealed that the amber and the co-occurring fossil foliage compressions of *Frenelopsis* Schenk (Cheirolepidiaceae) from the Lower Cretaceous of Spain are both characterized by the presence of phenolic abietane [42]. Although the totally absence of phenolic abietane in the Xixia amber may eliminate a significant contribution of Cheirolepidiaceae, the cheirolepidiaceous affinity cannot be completely excluded since compounds might be not extracted thus not detectable in the GC-MS analysis.

### Middle Miocene Zhangpu amber (Fig. 5; Table 2)

**Figure 5:**
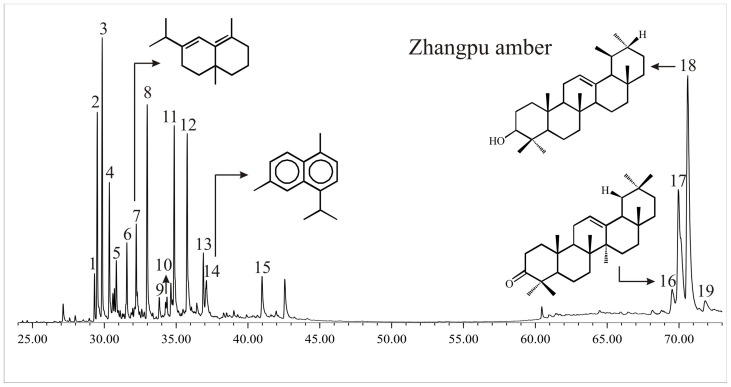
Total ion chromatogram of the middle Miocene Zhangpu amber from GC-MS analysis. The identified peaks are listed in Table 2.

The total ion chromatogram from the GC-MS analysis of the middle Miocene Zhangpu amber is given in the figure 5. The total extract of Zhangpu amber is characterized by sesquiterpenoids and triterpenoids. The major sesquiternoids are isoledene; 1H-3a,7-methanoazulene, octahydro-1,4,9,9-tetramethyl-(1α,3aα,4β,7α,8a); dehydro-ar-curcumene; δ-selinene; calamenene; methyl drimane and cadalene (Table 2). Triterpenoids such as α and β-amyrone; α and β-amyrin and hop-22(29)-en-3β-ol are abundantly present in the sample (Fig. 5; Table 2).

**Table 2:** Major compounds identified from the middle Miocene Zhangpu amber.

Peak number	Compound Name	Base peak	Molecular ion
1	Isoledene	105	204
2	Unknown C_15_sesquiterpenoid	81	206
3	Unknown C_15_sesquiterpenoid	95	206
4	1H-3a,7-Methanoazulene, octahydro-1,4,9,9-tetramethyl-(1α,3aα,4β,7α,8aβ)	163	206
5	Dehydro-ar- curcumene	119	204
6	Unknown C_15_sesquiterpenoid	191	206
7	δ-Selinene	161	206
8	Calamenene	159	202
9	Tetramethyl naphthalene	169	184
10	Methyl drimane	137	222
11	Unknown sesquiterpenoid	109	204
12	Methyl drimane+ Unknown sesquiterpenoid	137, 109	222, 204
13	Unknown C_15_sesquiterpenoid	81	206
14	Cadalene	183	198
15	Methyl drimane	137	222
16	β-amyrone	218	424
17	β-amyrin+ α-amyrone	218,218	426,424
18	α-amyrin	218	426
19	Hop-22(29)-en-3β-ol	189	426

The presence of amyrin and amyrone-based triterpenoids in the solvent extract of the Miocene Zhangpu amber indicates a contribution from angiosperms. Fossil resins are divided into four classes based on the molecular compositions [43]. Class I fossil resins are based primarily on polymers of labdatriene carboxylic acids, especially communic or ozic acids and are contributed by the Leguminosae. Fossil resins derived from conifers also belong to Class I [43]. Class II fossil resins are based on polymer of bicyclic sesquiterpenoid and triterpenoids hydrocarbons and are contributed by the family Dipterocarpaceae and a genus *Mastixia* Blume (Cornaceae) [44], [45]. Class III fossil resins with a polystyrene based structure are contributed by the family Hamamelidaceae. Class IV fossil resins, the botanical affinity of which is uncertain, have a cedrane polysesquiterpenoid based structure. The Zhangpu amber with a prominent distribution of cadalene-based sesquiterpenoid clearly belongs to Class II. Although trees of the genus *Mastixia* also produce resins of Class II, the occurrence of fossil winged fruits of Dipterocarpaceae in the Fotan Group of Zhangpu [26], [27] confirms a Dipterocarpaceae origin of the Zhangpu amber.

Dipterocarpaceae are a tropical tree family that dominate the emergent canopy of most lowland rain forests in the Southeast Asia [46]. The family comprises three subfamilies: Dipterocarpoideae in the Asian tropics and Seychelles; Pakaraimoideae restricted to the Guyana and Venezuela of tropical South America; and Monotoideae in tropical Africa, Madagascar and Southeast Colombia [46]. Molecular phylogenetic and biogeographic studies indicate that the family had an ancient Gondwanan origin and arrived in Asia after the establishment of the land connection between the Indian and Asian plates [14], [47], [48]. This hypothesis was supported by the earliest fossil record of the family which is from the lower Eocene of India [14]. Among the family only trees of the Asian subfamily Dipterocarpoideae are highly resinous whereas the Pakaraimoideae and Monotoideae lack resin ducts [46]. Amber derived from the Dipterocarpaceae has been reported from the Eocene and Miocene of India [13], [14], [15], [49] and the Eocene of Vietnam [50]. The middle Miocene Zhangpu amber represents the northernmost distribution of Dipterocarpaceae-derived amber and this discovery corroborates the existence of a dipterocarp forest in Zhangpu of Southeast China and the northward movement of the tropical family during the Mid-Miocene Climatic Optimum [26], [27]. Today the Dipterocarpaceae is absent in the Zhangpu area, which today has a typical subtropical monsoon climate and subtropical evergreen broadleaved forests [51].

## Conclusions

In this paper we studied the terpenoid compositions and botanical origins of the Late Cretaceous and middle Miocene amber from China by GC–MS analysis. This work represents the first detailed biomarker study for amber from East Asia. The Late Cretaceous Xixia amber is characterized by sesquiterpenoids, abietane and phyllocladane type diterpenoids, but lacks phenolic abietanes and labdane derivatives. This suggests a significant contribution of Araucariaceae to the Xixia amber. Although no plant fossils have been reported in the Late Cretaceous red bed of Xixia, paleobotanical records indicate that the Araucariaceae, which is mainly distributed in the Southern Hemisphere today, did occur in the Late Cretaceous of the mid-latitude regions of the Northern Hemisphere.

The total extracts of the middle Miocene Zhangpu amber contain amyrin and amyrone-based triterpenoids, and cadalene-based sesquiterpenoids. These compounds are characteristic for dammar resins that are produced by trees of the tropical family Dipterocarpaceae. The Dipterocarpceae origin of the Zhangpu amber is also corroborated by the co-occurring fossil winged fruit of this family. All evidence suggests the presence of a dipterocarp forest in the Southeast China during the middle Miocene.
